# Nociceptive Afferents to the Premotor Neurons That Send Axons Simultaneously to the Facial and Hypoglossal Motoneurons by Means of Axon Collaterals

**DOI:** 10.1371/journal.pone.0025615

**Published:** 2011-09-29

**Authors:** Yulin Dong, Jinlian Li, Fuxing Zhang, Yunqing Li

**Affiliations:** Department of Anatomy and Histology and Embryology, and K. K. Leung Brain Research Centre, the Fourth Military Medical University, Xi'an, China; Harvard University, United States of America

## Abstract

It is well known that the brainstem premotor neurons of the facial nucleus and hypoglossal nucleus coordinate orofacial nociceptive reflex (ONR) responses. However, whether the brainstem PNs receive the nociceptive projection directly from the caudal spinal trigeminal nucleus is still kept unclear. Our present study focuses on the distribution of premotor neurons in the ONR pathways of rats and the collateral projection of the premotor neurons which are involved in the brainstem local pathways of the orofacial nociceptive reflexes of rat. Retrograde tracer Fluoro-gold (FG) or FG/tetramethylrhodamine-dextran amine (TMR-DA) were injected into the VII or/and XII, and anterograde tracer biotinylated dextran amine (BDA) was injected into the caudal spinal trigeminal nucleus (Vc). The tracing studies indicated that FG-labeled neurons receiving BDA-labeled fibers from the Vc were mainly distributed bilaterally in the parvicellular reticular formation (PCRt), dorsal and ventral medullary reticular formation (MdD, MdV), supratrigeminal nucleus (Vsup) and parabrachial nucleus (PBN) with an ipsilateral dominance. Some FG/TMR-DA double-labeled premotor neurons, which were observed bilaterally in the PCRt, MdD, dorsal part of the MdV, peri-motor nucleus regions, contacted with BDA-labeled axonal terminals and expressed *c-fos* protein-like immunoreactivity which induced by subcutaneous injection of formalin into the lip. After retrograde tracer wheat germ agglutinated horseradish peroxidase (WGA-HRP) was injected into VII or XII and BDA into Vc, electron microscopic study revealed that some BDA-labeled axonal terminals made mainly asymmetric synapses on the dendritic and somatic profiles of WGA-HRP-labeled premotor neurons. These data indicate that some premotor neurons could integrate the orofacial nociceptive input from the Vc and transfer these signals simultaneously to different brainstem motonuclei by axonal collaterals.

## Introduction

The trigeminal motor nucleus (V), facial nucleus (VII) and hypoglossal nucleus (XII) mainly administrate the orofacial muscle, such as jaw, lips, tongue, palate, pharynx, esophagus, larynx, diaphragm and other respiratory muscles and are involved in the orofacial coordinated activities. Electrophysiological and morphological studies demonstrated that numerous premotor neurons were distributed principally in the pontomedullary reticular formation (RF), raphe nucleus, trigeminal sensory complex, parabrachial region between the cerebral motor cortex and the brainstem orofacial motonucleiand precisely coordinated for executing complex muscular functions [1]–[4]. Besides the movements induced by cerebral motor cortex, previous electromyographic (EMG) recording indicated the existence of reflex activities of the orofacial muscles, including blink reflex (BR), corneal reflex (CR), levator palpebrae inhibitory reflex (LPIR), jaw reflex (JR) in human or animals [5], [6]. The peripheral orofacial stimuli, such as nociceptive stimulation, induced the orofacial muscles reflex responses and corresponding EMG changes after decerebration [7], [8], which indicated that the local circuits exist in the brainstem. These circuits can integrate the peripheral stimuli, transfer them to the brainstem motoneurons and accomplish the reflexes activity. Further studies on EMG showed that most of the reflexes might be induced by peripheral nociceptive stimuli and was especially expressed in the R2 and R3 phases of EMG recording [9]–[11] indicating the existence of the premotor neurons on the orofacial nociceptive reflex (ONR) pathways. However, so far, few investigations were made into the composition of ONR local transmission pathways and the morphological distribution of the premotor neurons on the pathways.

The caudal spinal trigeminal nucleus (Vc) is involved in conveying nociceptive input from the skin of the face, lips, tooth pulp, oral and nasal cavities, cornea, and so on. Previous studies identified that the Vc was not only the region where the trigeminal primary afferents terminated but also the potential structure underlying the local pathways of the ONR [12], [13]. Anatomic studies reported that the Vc projecting neurons sent their axonal collaterals to the pontomedullary RF, the trigeminal sensory complex, the raphe nucleus complex, etc. [14], [15]. Moreover, morphological [3] studies have identified that the distribution of the premotor neurons involving in the coordinated movement of the orofacial muscles largely overlapped with those of Vc projecting afferents fibers and terminals in the brainstem. Therefore, it was postulated that these premotor neurons may be involved in the ONR [16]. However, there was few morphological evidence to support this postulation until now.

In addition, most orofacial motor nuclei, such as the VII and XII, are largely implicated in synchronizing various oral movements and performing ONR. By using fluorescent retrograde double-labeling method, the previous morphological studies showed that the distribution of the premotor neurons related to the VII or XII was overlapped [17], and single premotor neuron projected bilaterally to the V, VII or XII and innervated two of these three motor nuclei by their branched axons. These findings indicated that the bifurcating premotor neurons probably subserved the coordination of orofacial movements required for the complex oral motor behaviors [18], [19]; Moreover, these researches gave rise to a hypothesis that single premotor neuron may be involved in the different ONR simultaneously. However, there still lacks evidence whether or not axonal terminals from the Vc synapse directly on those premotor neurons which transmit orofacial nociceptive information simultaneously to the VII and XII, and then further accomplish the complicated and coordinated oral nociceptive reflex (NR).

Our present studies aimed at (1) detecting whether the projection fibers from Vc might make synaptic contact to the premotor neurons projecting to VII or XII by using a double-labeled technique of biotinylated dextran amine (BDA) anterograde tracing combined with Fluoro-gold (FG), tetramethylrhodamine-dextran amine (TMR-DA) or wheat germ agglutinated horseradish peroxidase (WGA-HRP) retrograde transport; (2) examining whether the premotor neurons sending collaterals to the VII and XII simultaneously receive afferents from the Vc; (3) further identifying the nociceptive premotor neurons in the brainstem NR pathways in which the immunoreactivity for Fos was induced by subcutaneous injection of formalin into the upper and lower lips.

## Materials and Methods

A total of 35 adult male rats (Sprague-Dawley; China SH, Xi'an, People's Republic of China) weighing 250–300 g were housed in a standard laboratory condition (artificial light cycle 12 h on/12 h off) with food and water *ad libitum* were used. All the experimental protocols and animal care were approved by the Institutional Animal Care and Use Committees of the Fourth Military Medical University of China prior to the onset of experiments (Permit number: 10001).

All of rats were divided into four groups (Table 1). Thirty of them were used for light microscopic study (three groups, n = 10 for each group), and the remaining 5 rats were used for electron microscopic study (the fourth group). In the first and second groups, BDA, an anterograde tracer, was injected into the Vc and FG, a retrograde tracer, was injected into the VII or XII at the same side to observe the relationship between the Vc projecting fibers and the premotor neurons of the VII or XII. In the third group, BDA was injected into the Vc combined with FG and TMR-DA which were injected into VII and XII at the same side, respectively (Table 1). In some rats of the first three groups, Fos was induced in the nociceptive neurons by subcutaneous injection of formalin into the upper and lower lips. Electron microscopy was used to examine whether the projection fibers from the Vc make synapses on the premotor neurons projecting to the VII or XII by a double-labeled method after the BDA injection into the Vc, WGA-HRP into the VII or XII (Table 1).

**Table 1:** Experimental Cases.

	n	purpose	tracer injection sites	formalin stimulation (n/group)
Group 1	10	light microscope	FG injected into the VII and BDA into the Vc	4
Group 2	10	light microscope	FG injected into the XII and BDA into the Vc	4
Group 3	10	light microscope	FG injected into the VII, TMR-DA into the XII and BDA into the Vc	4
Group 4	5	electron microscope	WGA-HRP injected into the VII (2 animals) or the XII (3 animals) and BDA into the Vc	no

### Anterograde-labeling tracer injection

BDA was used as an anterograde tracer to label axonal terminal of the Vc projecting neurons for epifluorescence, confocal laser scanning microscopic and electron microscopic studies. The animals were anesthetized by sodium pentobarbital (40 mg/kg, i.p.) until no limb-withdrawal reflex was elicited by pinching the hind paw. They were placed on a stereotaxic frame, and the cisternal cavity of the caudal medulla oblongata was surgically exposed. An approximate volume of 0.2 µl of a 10% solution of BDA (10,000 MW, D1956, Molecular probes, Eugene, OR, USA) dissolved in 0.05 M phosphate-buffered saline (PBS, pH 7.4) was injected into the Vc by pressure through a glass micropipette (internal tip diameter = 15–25 µm) which was attached to a 1-µl Hamilton microsyringe.

### Retrograde-labeling tracer injection

For light microscopic study in the first two groups, 4% of FG (80014, Biotium, Hayward, CA, USA) dissolved in 0.05 M PBS was iontophoresed into the VII or XII, respectively, on the side ipsilateral to the side of the BDA injection into the Vc with 7 µA positive current pulses (7 s on/7 s off) for 15 minutes after BDA injection. In the third group, 4% of FG was ipsilaterally iontophoresed into the VII after BDA injection, and then an approximate volume of 0.05–0.1 µl of a 10% TMR-DA (D3308, 3000 MW, anionic, lysine fixable; Molecular Probes, Eugene, OR) dissolved in 0.1 M citrate-NaOH (pH 3.0) [20] was injected into the XII ipsilaterally by pressure, as described above. For electron microscopic study in the fourth group, seven days after the injection of BDA, a solution of 2% WGA-HRP (836500, Toyobo, Osaka, Japan) was ipsilaterally iontophoresed into the VII or XII, respectively, with 2 µA current pulses (5 Hz) for 10–15 minutes.

### Orofacial formalin injection

After the FG or/and TMR-DA injection, the rats were allowed to survive for 7 days and then they were killed by transcardial perfusion. Two hours before the perfusion, the 12 rats (4/group) in the first three groups were anesthetized with ethyl ether. Then a volume of 0.2 ml of 1.5% formalin dissolved in physiological saline was injected subcutaneously into the upper and lower lips ipsilateral to the surgery using a 25-gauge needle. The other rats were used as normal control (n = 6, 2/group) or experimental control (n = 12, 4/group). The experimental control received a single injection of 0.2 ml of physiological saline instead of the formalin solution.

### Immunohistochemical studies

After surviving for 7 days, the rats in the first three groups were deeply anesthetized by sodium pentobarbital (100 mg/kg, i.p.) and perfused transcardially with 100 ml of a solution consisting of 0.9% saline in 0.05 M phosphate buffer (PB, pH 7.4), followed by a volume of 500 ml of 0.1 M PB containing 4% paraformaldehyde and 75% (v/v)-saturated picric acid. The brains were removed and placed in 0.1 M PB containing 30% (w/v) sucrose at 4°C for 24 h. Subsequently, the brainstems were serially cut into transverse sections 30 µm thick on a freezing microtome. The sections were divided into five series and collected into 0.05 M PBS (pH 7.4).

The first series of every group was for observation of the injection site of the VII or/and XII, the distribution of retrograde labeled neurons projecting to the VII or XII (Group 1 and 2), and the retrograde double-labeled neurons projecting to both the VII and XII (Group 3) by an epifluorescence microscope (BX-60; Olympus, Tokyo, Japan).

The second series of every group was processed for visualization of the injection site of Vc and the distribution of BDA-labeled fibers in the brainstem. The sections were incubated in 0.5% Triton X-100 in 0.05 M PBS (pH 7.6) overnight prior to incubation in the fluorescent isothiocyanate (FITC) -labeled avidin D (1∶200, A-2001, Vector Laboratories, Burlingame, CA, USA) at room temperature for 2 h. After the incubation, all the sections were rinsed in 0.05 M PBS, mounted onto gelatin-coated glass slides, air-dried, cover-slipped by a mixture of 50% (v/v) glycerin and 2.5% (w/v) triethylene diamine (anti-fading agent) in 0.05 M PBS. Then the injection sites and distribution of BDA-labeled fibers were observed by the epifluorescence microscope.

The third series of Groups 1 and Group 2 was processed for double-labeling study of FG-labeled neurons and BDA-labeled fibers and terminals. The sections were incubated at room temperature first with rabbit anti-FG IgG (1∶5000, AB153, Chemicon, Temecula, CA, USA) overnight and then with a mixture of 10 µg/ml FITC-labeled donkey anti-rabbit IgG antibody (AP182F, Chemicon), and 10 µg/ml Texas Red (TR)-labeled avidin D (A-2006, Vector Laboratories) for 4 h. The incubation medium used for the primary antibodies was 0.05 M PBS (pH 7.4) containing 2% normal donkey serum (NDS), 0.5% Triton X-100, 0.05% sodium azide (NaN_3_) and 0.25% λ-carrageen (NDS-PBS). The incubation medium for the secondary antibodies was 0.05 M PBS (pH 7.4) containing 0.5% Triton X-100.

The third series of Group 3 was used to observe the triple-labeling in FG, TMR and BDA. Briefly, the sections were incubated at room temperature first with rabbit anti-FG IgG and guinea pig (Gp) anti-TMR IgG (1∶2000) [20] overnight and then with a mixture of 10 µg/ml FITC-labeled donkey anti-rabbit IgG (AP182F, Chemicon), 10 µg/ml rhodamine (Rh)-labeled goat anti-Gp IgG (AP108R, Chemicon) and 10 µg/ml indodicarbocyanine (Cy5)-labeled avidin D (AQ132F, Chemicon) for 4 h. The incubation mediums were similar with those in the third series of Group 1 and 2.

The fourth series of Group 1 and Group 2 was used to identify the contact between the BDA-labeled fibers and retrograde double-labeled neurons with FG and Fos. Briefly, the sections were incubated firstly with a mixture of rabbit anti-FG IgG, mouse anti-Fos IgG (1∶1000, SC-413, Santa Cruz, CA, USA) at room temperature for 24 h and at 4°C for 24 h, and then with a mixture of FITC-labeled donkey anti-rabbit IgG antibody, Cy5-labeled goat anti-mouse IgG (1∶200, AP127S, Chemicon) and Rh-labeled avidin D for 4 hours. The incubation mediums were similar to those in the third series. The fourth series of Group 3 was used for the immunofluorescence histochemical study of the coexistence among the FG, TMR retrograde doubled-labeled neurons and Fos. The process was similar to that in the third series of Group 1 and 3 except for substitution of TMR-immunohistochemistry for BDA-immunohistochemistry. For controls, some sections were also processed as above but with omission of the first anti-Fos IgG, which resulted in no staining for the Fos protein.

The sections in the fifth series of every group were mounted onto gelatin-coated glass slides and then stained with 1% cresyl violet. Large projection drawings of these cresyl-violet-stained sections were then prepared, and the location of FG or/and TMR-DA-labeled neuronal cell bodies were plotted on the projection drawings with and aid of a camera lucida attachment. Subsequently, the data were reconstructed onto projection drawings of sets of serial sections.

### Electron microscopic study

The rats in Group 4, which survived seven days after the injection of BDA, were allowed to survive for additional 48 h following WGA-HRP injection. These rats were deeply reanesthetized by injection of an overdose of sodium pentobarbital (100 mg/kg, i.p.), and then perfused transcardially with 100 ml of 0.025 M PBS(pH 7.4), followed by 500 ml of a fixative consisting of 4% paraformaldehyde and 0.1% glutaraldehyde in 0.1 M PB (pH 7.4). The brains were removed and stored in 0.1 M PB (pH 7.4) containing of 4% paraformaldehyde at 4°C for 2–4 h.

Serial sections of the pons and medulla were cut transversely on a vibratome (Microslicer DTM-1000; Dosaka EM, Kyoto, Japan) at 50 µm thickness. Tissue sections were processed for the histochemical demonstration of WGA-HRP by using tetramethylbenzidine-sodium tungstate (TMB-ST) method [21] and the WGA-HRP reaction products were intensified with DAB/Cobalt/H_2_O_2_ solution [22]. The sections were incubated in 20% normal goat serum (Vector) in 50 mM Tris-buffered saline (TBS, pH7.4) for 1–2 h followed by the incubation in 1∶50 avidin-biotin-peroxidase (ABC) complex (AP182S, Vector) at room temperature for 6 h and then at 4°C 6 h. The BDA labeling was revealed with diaminobenzidine (DAB)-nickel-ammonium sulfate method, followed by routine EM processing [23].

### Images acquisition and handling

The BDA-labeled fibers and FG/TMR-labeled neurons were scrutinized and counted under the epifluorescence microscope (BX-60, Japan) with appropriate filters for TMR (excitation 540–552 nm; emission≥575 nm), FG (excitation 350–395 nm; emission 430 nm) and FITC or DTAF (excitation 450–490 nm; emission 515–565 nm). Digital images of fluorescent labeling were taken by a DP-70 CCD camera and software (Olympus Optical Co., Inc., LTD, Japan) connected to a computer for permanent documentation. Two separate images from the same field were captured with two different filters corresponding to the specific combination of the tracers.

The immunofluorescence histochemical results were observed by a confocal laser-scanning microscope (CLSM, FV1000, Olympus, Tokyo, Japan) by using laser beams of 490, 590 and 640 nm with the appropriated emission filter for FITC (520 nm), Rh (615 nm) and Cy5 (705 nm). Then the digital images were arranged and modified (15–20% contrast enhancement) in software Tiff files. The ultrastructures were observed by an electron microscope (CM100; Philips, Eindhoven, The Netherlands).

## Results

### 1. Distribution of premotor neurons projecting to the VII or/and XII

**Figure 1:**
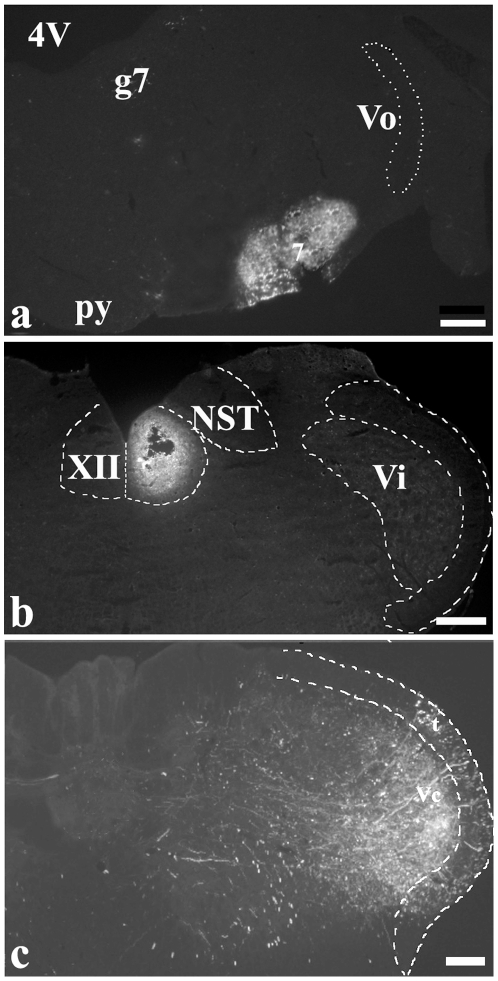
Injection site. Fluorescent photomicrographs of a section through the brainstem, showing the sites of FG centered on the facial nucleus (VII) (a), hypoglossal nucleus (XII) (b) and BDA on the caudal spinal trigeminal nucleus (Vc)(c). Scale bars = 250 µm.

The cases in which injection sites were centered in the VII (Fig. 1a) or/and XII (Fig. 1b) in the rats of Group 1, 2 and 3 without diffusion to the overlying reticular formation were taken for the further analysis.

#### Retrogradely labeled neurons: FG injection into the VII or XII

In all successful cases of the first two groups, most FG retrogradely labeled neuronal cell bodies were medium (15–34 µm), triangular and multipolar in shape. There were also a few large (≥34 µm) multipolar, small (≤15 µm) fusiform or circular neuronal cell bodies by chance.

In the case of the VII injection, the retrogradely labeled neurons were observed throughout the brainstem with a level of caudal segment prevalence, the distribution pattern of which was generally consistent with that described in our previous study [24], mainly in the lateral, dorsal and the ventral parts of the RF around the raphe magnus and the gigantocellular reticular nucleus pars alpha (Giα), bilaterally with a slight ipsilateral dominance. A number of FG-labeled neurons were also seen in the Vi (interpolar spinal trigeminal nucleus), Vo (oral spinal trigeminal nucleus), Vp (principal sensory trigeminal nucleus), the lateral and medial parabrachial nuclei (LPB and MPB), the nucleus of Kölliker-Fuse (KF), and nucleus of the solitary tract (NST) and peri-motor nuclei, such as the supratrigeminal nucleus (Vsup), the juxtatrigeminal region, etc. Besides, a few FG-labeled neurons were found in the XII. A small number of FG-labeled neurons were also observed in the intermediate zone of the upper and middle cervical segment of the spinal cord.

In the case of the XII injection, most FG-labeled neuronal cell bodies were observed bilaterally in the pontomedullary RF with a slight dominance on the side ipsilateral to the injection site. The pattern of distribution of these labeled neuronal cell bodies in the pontomedullary RF was the same as that after FG injection into the VII. Moreover, a few retrogradely FG-labeled neuronal cell bodies were observed in the superficial layer of the Vc ipsilateral with the injection site.

#### Retrograde doubly labeled neurons: FG injection into the VII and TMR-DA into the XII

**Figure 2:**
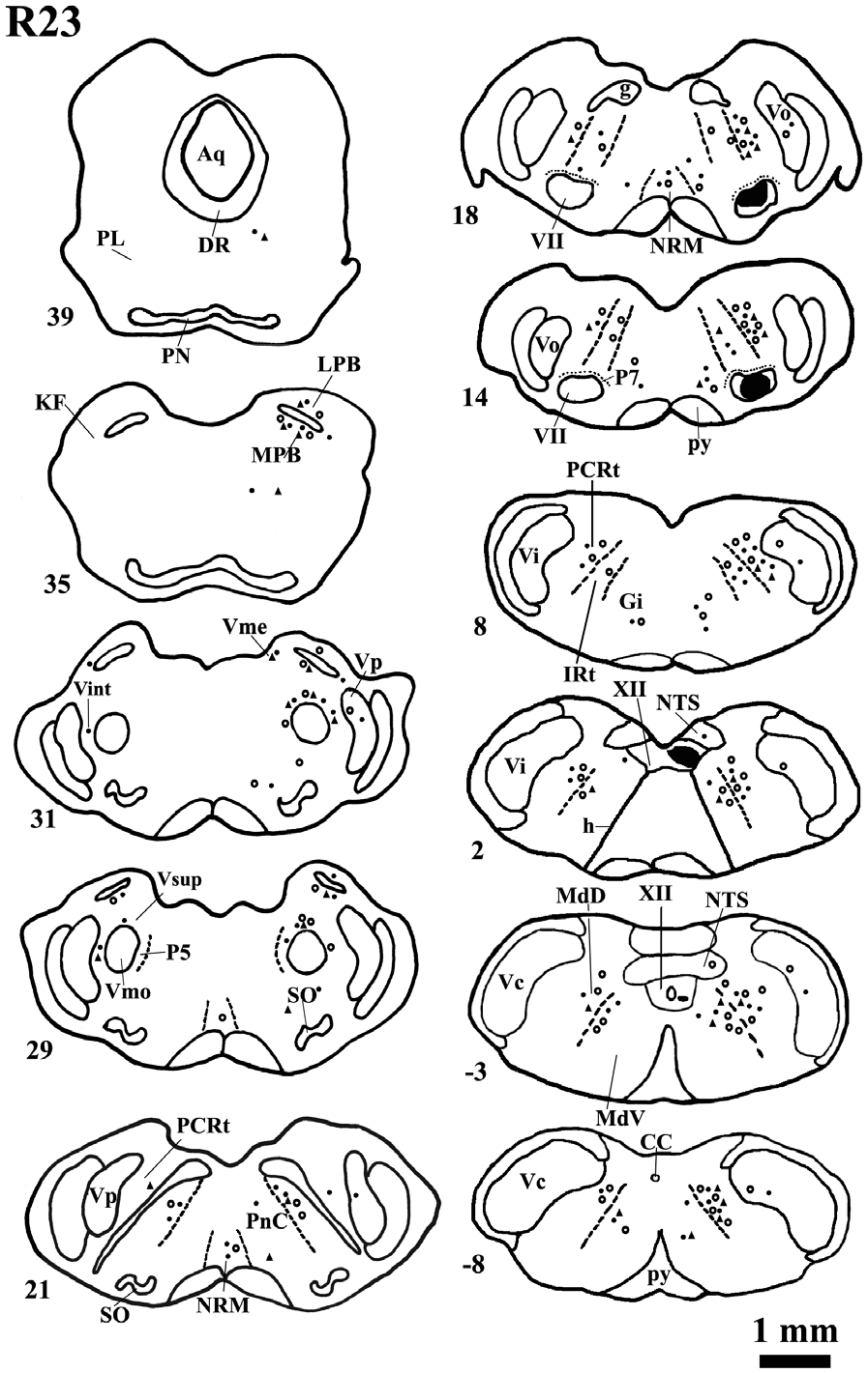
The distribution pattern of the retrograde FG/TMR-DA double-labeled neuron. Projection drawing of transverse section through the brainstem of R23, showing the distribution pattern of the retrograde double-labeled neuronal cell bodies with FG injection into the VII and TMR-DA into the ipsilateral XII. The injection sites are blackened. Neuronal cell bodies single-labeled with FG, TMR-DA, or those dually labeled with FG/TMR-DA are represented by filled circles, open circles or filled triangles. The filled circles and open circles are plotted in one-to-three fashion, whereas the filled triangles are one-to-one fashion. Scale Bar = 1 mm.

**Figure 3:**
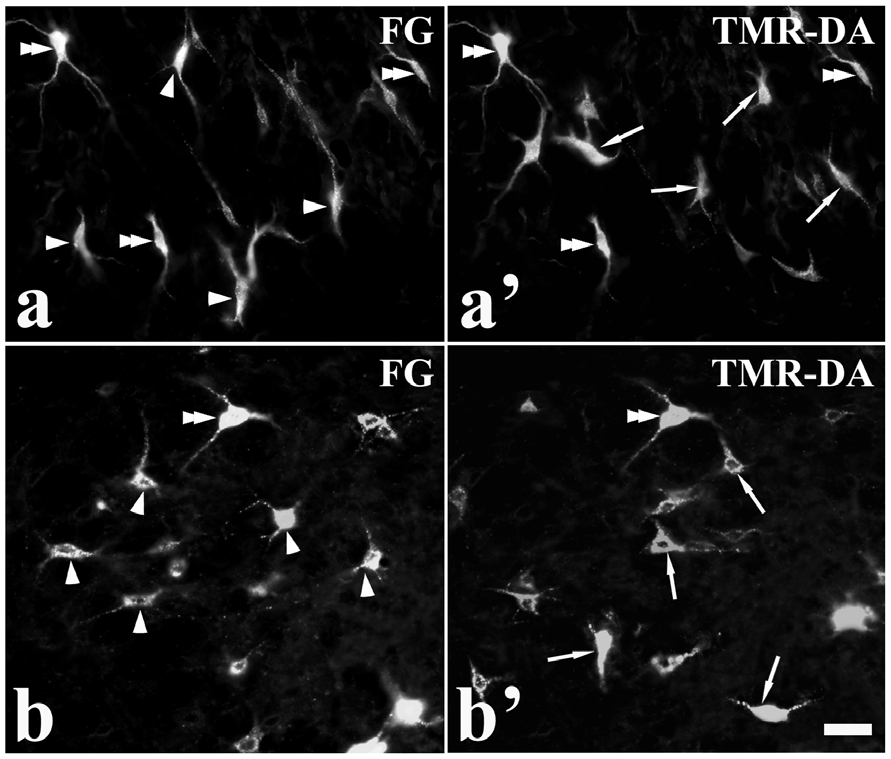
FG/TMR-DA double-labeled neurons. a–b′, Fluorescent photomicrographs showing FG (a and b) and TMR-DA (a′ and b′) retrograde-labeled neurons in the brainstem reticular formation after FG injection into the VII and TMR-DA ipsilaterally into the XII. Double-arrowheads indicate FG/TMR-DA double-labeled neurons, arrowheads and arrows indicate FG or TMR-DA single-labeled neurons, respectively. a and b were taken from the parvicellular reticular formation and the dorsal medullary reticular formation, respectively. The fields of a–b corresponds with that of a′–b′. Scale bar = 35 µm.

After FG and TMR-DA were injected into both the VII and XII at the same side, respectively, FG/TMR-DA double-labeled neuronal cell bodies were observed bilaterally in the brainstem with an ipsilateral dominance (Fig. 2, 3). The most dense FG/TMR-DA double-labeled neurons were mainly located in the pontomedullary RF, PB, the regions around the Vmo or VII, including Vsup, ventral RF of Vmo, and Gi, etc. In Group 3, the numbers of doubly labeled neurons were counted in every fifth section in each series of serial sections of the lower brainstem. The number of FG/TMR-DA double labeled neurons in one rat ranged from 33 to 47 in pontomedullary RF, 2 to 4 in PBN and from 4 to 6 in regions around the Vmo. Approximately 3.2–7% of the total retrograde labeled premotor neurons were doubly labeled with FG/TMR-DA in the brainstem (Table 2).

**Table 2:** Double-labeled neurons in some regions of the brainstem after injections of FG and TMR-DA into the facial and hypoglossal nucleus, respectively, of the rats in the group 3.

rat number	pontomedullary RF			PBN			regions around the Vmo		
	FG	TMR	FG/TMR (%)	FG	TMR	FG/TMR(%)	FG	TMR	FG/TMR (%)
R23	394	283	46(6.4)	49	23	3(4.0)	67	53	5(4.0)
R24	365	257	39(5.9)	43	19	3(4.6)	63	41	4(3.7)
R26	325	253	33(5.4)	43	18	2(3.2)	58	44	4(3.8)
R27	386	288	47(6.5)	51	27	4(4.9)	69	47	6(4.9)
R29	369	264	41(6.1)	47	24	4(5.3)	65	44	4(3.5)
Total	1839	1345	206(6.1)	233	111	16(4.4)	322	229	23(4.0)

In each rat, the numbers of FG-, TMR-single-labeled and FG/TMR-double-labeled neurons were counted in the pontomedullary reticular formation (RF), parabrachial nucleus (PBN) and the regions around the Vmo on both sides. And then, the ratio of FG/TMR-double-labeled neurons to total retrograde-labeled neurons (FG+TMR+FG/TMR) was calculated in each region. Cell counts were obtained from every fifth section of a series of serial sections of 30 µm thickness.

### 2. Contact between BDA-labeled fibers and FG-single-labeled or FG/TMR double-labeled premotor neurons

#### 2.1 The fibers from the Vc contacted FG-single-labeled or FG/TMR double-labeled neurons projecting to the VII or/and XII

**Figure 4:**
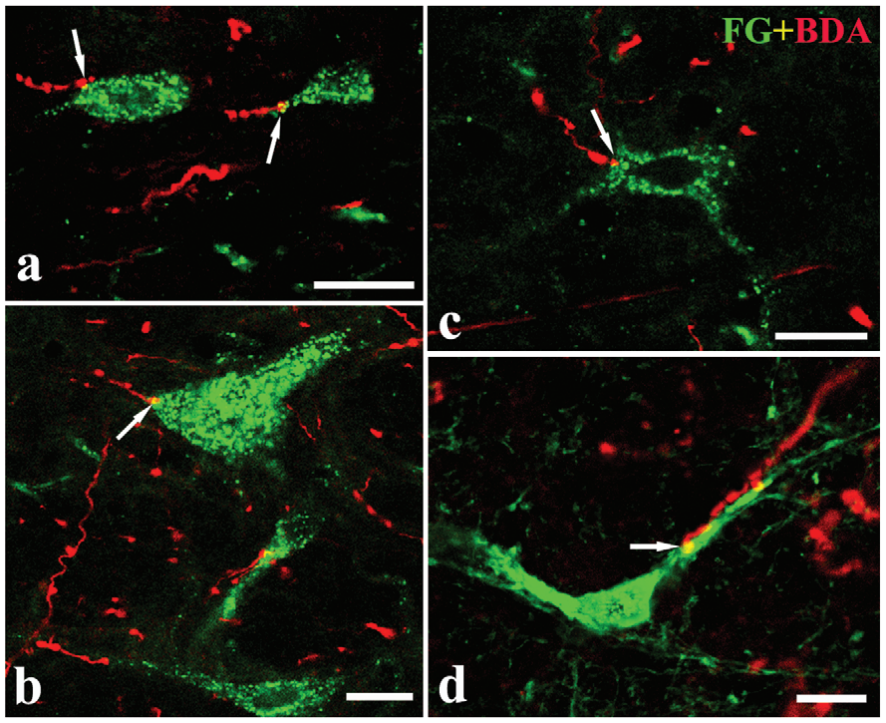
The close appositions between axonal terminals of the Vc and the premotor neurons of the VII or XII. Digital images showing the close appositions (arrows) between axonal terminals of the Vc neurons and FG retrograde-labeled dendrites (a, d) or neuronal cell bodies (a, b and c) of the premotor neurons in the brainstem reticular formation after BDA injection into the Vc and FG into the VII (a, b) or XII (c, d). a and c were taken from the parvicellular reticular formation, b from the supratrigeminal nucleus, d from the dorsal medullary reticular formation. Scale bars = 20 µm in a, b and d, 18 µm in c.

Double labeling method of BDA anterograde tracing combined with FG retrograde transport examined if the fibers and terminals of the trigeminal nociceptive afferents contacted the premotor neurons which send their axons to the VII or XII by CLSM. After BDA injected into the Vc (Fig. 1C), the BDA-labeled fibers were observed, bilaterally with an ipsilateral dominance, from the rostral to ventral of the brainstem, and a widely scattered distribution of the labeled fibers and terminals was observed from the pontomedullary RF in the present study. The FG-labeled premotor neurons were green while the BDA-labeled Vc projecting terminals were red. A large number of BDA-labeled fibers and terminals surrounded FG-labeled premotor neurons and apposed to both the soma and dendritic arborizations of them (Fig. 4). The appositions between BDA-labeled fibers and FG-labeled premotor neurons were mainly distributed throughout the bilateral brainstem RF with an ipsilateral predominance of injection site. In the caudal part of the medulla oblongata, these close contacts between BDA-labeled fibers and FG-labeled neurons were mainly distributed in the medullary RF, such as MdD and the dorsal part of MdV, in which the highest density of the BDA anterograde labeling and FG retrograde labeled premotor neurons was found. From the level of the fourth ventricle opening completely, these close contacts were located densely in the dorsal part of PCRt, which continued directly from the dorsal part of the medullary reticular field in the caudalmost medulla oblongata and IRt. In addition, moderate appositions between them were observed in the regions around Vmo, PBN; however, there were a few appositions in the ventral part of the pontine reticular formation.

**Figure 5:**
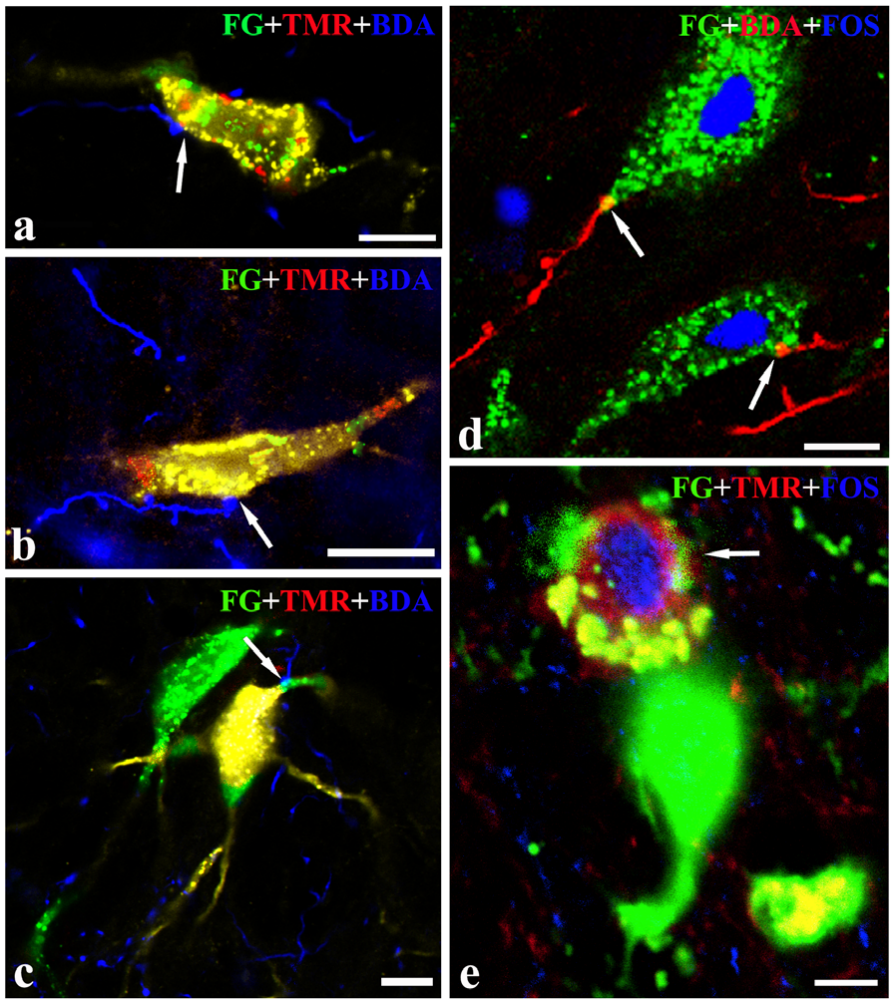
The nociceptive projections from the Vc making close appositions to the premotor neurons which send axons simultaneously to the VII and XII by means of axon collaterals. a, b and c, digital images showing the close appositions (arrows) between axonal terminals of the Vc neurons and FG/TMR retrograde-double-labeled neuronal cell bodies (a, b) or dendrite (c) after FG injection into the VII, TMR-DA into the XII and BDA into ipsilateral Vc. Immunoreactivities for FG, Fos and BDA are visualized with FITC (green), Cy5 (blue), and Rh (red), respectively. d, BDA-labeled axonal fibers and terminals making close apposition to dendrite or neuronal cell bodies of FG-labeled premotor neurons expressing Fos-like immunoreactivities. e, FG/TMR/Fos triple-labeled neurons after FG injection into the VII, TMR-DA into the XII combined with the formalin ipsilaterally subcutaneous stimulation. a, b, d and e are taken from the ventral medullary reticular formation, and c is from the parvicellular reticular formation. Scale bars = 10 µm in a, c and d; 15 µm in b and 5 µm in e.

To examine whether the premotor neurons receiving trigeminal nociceptive afferents sent their bifurcating axon simultaneously to the VII and XII, the triple-labeling method of BDA anterograde tracing combined with FG and TMR-DA retrograde transport was applied. After FG and TMR-DA were injected into the VII and XII, respectively, and BDA was injected into the Vc, the appositions between BDA-labeled terminals and FG/TMR double-labeled premotor neurons were observed in pontomedullary RF, mainly including the ventral part of Vmo, PCRt, MdD and MdV, in which the highest density of the double-labeled premotor neurons was distributed(Fig. 5a–c).

#### 2.2 Expression of Fos protein in the FG single-labeled or FG/TMR double-labeled premotor neurons

To further identify whether the FG or FG/TMR double-labeled premotor neurons were located in the ONR pathway, Fos expression (blue color) was observed at the fourth section in each series of serial sections of Group 1, 2 and 3 in which FG or FG/TMR-DA were injected into the VII or/and XII and BDA into the Vc (Table 1). Numerous neurons expressing Fos proteins after formalin injection to lips were mainly distributed in Vc, the transition level from Vc to Vi, the cervical spinal cord, NST and PBN. Moreover, the Fos-like immunoreactive (LI) neurons were found in the reticular formation, such as PCRt, MdD, MdV with ipsilateral predominance. In the Group 1 and 2 which were injected FG into the VII or XII, there were some Fos positive neurons which were labeled retrogradely with FG simultaneously. The FG and Fos double-labeled neurons were mainly located in the ventrolateral part of the MdV and the ventral part of the PCRt. In addition, there were double-labeled neurons observed in P5. For each group, we selected two successfully operated animals, counted the number of Fos positive and Fos/FG double-labeled neurons and then calculated the ratio of the double-labeled neurons to the Fos positive neurons (see table 3). In one rat of Group 1 successfully operated, the number of Fos positive neurons were 94 in the MdV; approximately 9.6% of these Fos-labeled neurons were FG retrogradely labeled. And in one rat of Group 2, approximately 8.1% of the Fos-labeled neurons were FG retrogradely labeled in the MdV. Under the CLSM, some BDA-labeled fibers were observed to make close appositions to FG/Fos double-labeled premotor neurons (Fig. 5d). After FG, TMR were injected into the VII and XII, respectively, and BDA into the Vc, there were some FG/TMR double-labeled premotor neurons expressed Fos-LI simultaneously (Fig. 5e) in the pontomedullary RF, especially in the MdD and PCRt. The number of FG/TMR/Fos triple-labeled neurons ranged from 2 to 5 in the RF in the rats of Group 3.

**Table 3:** Counts of Fos positive neurons which were labeled retrogradely with FG injected into the VII (Group 1) or XII (Group 2).

Group	Animal number	MdV		PCRt	
		Fos	FG+Fos (%)	Fos	FG+Fos (%)
1	No. 4	94	9(9.6)	158	12(7.6)
	No. 9	87	5(5.7)	181	9(5.0)
2	No. 13	123	10(8.1)	104	7(6.7)
	No. 15	148	9(6.1)	99	4(4.0)

In each rat, the total number of Fos positive neurons were counted in the MdV and PCRt. Of these Fos-labeled neurons, the numbers of FG retrogradely labeled ones (FG+Fos) were also counted. The ratio of FG+Fos to total Fos (%) was calculated in each region. Cell counts were obtained from every fifth section of a series of serial sections of 30 µm thickness.

However, there was no FG/Fos double-labeled neurons observed in the normal and control groups without injection with formalin.

### 3. Synapses between BDA-labeled terminals from Vc and WGA-HRP- labeled premotor neurons projecting to the VII or XII

Synaptic connections of projection fibers from Vc with the premotor neurons which send their axons to the VII or XII were examined by electron microscopy with a double-labeled method after injection of BDA into the Vc, WGA-HRP into the VII or XII. After WGA-HRP was injected into the VII or XII, the distribution pattern of retrogradely WGA-HRP-labeled neurons was similar to that FG injected into the VII or XII.

**Figure 6:**
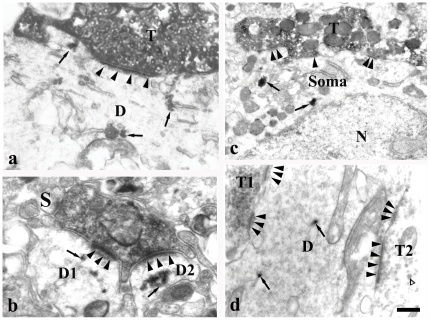
Electron micrograph showing BDA-labeled terminals and WGA-HRP-labeled soma and dendrites in the brainstem of a rat. WGA-HRP was injected into the VII of the rat and BDA into its ipsilateral Vc. Note: BDA-labeled terminals were identified by fine granular and homogeneously distributed electron dense reaction product with the dense distribution on the membrane of the synaptic vesicles or outer membrane of the mitochondria,and most synaptic vesicles are clear spherical in their terminal. HRP-labeled postsynaptic components exhibited highly electron dense, clumps of crystalline or amorphous material (arrows) in the cytoplasm and dendritic profiles. BDA-labeled S-type terminals (T) containing sphere vesicles make asymmetric synapses with dendrites (D) of HRP-labeled premotor neurons (a). BDA-labeled terminal forms asymmetric synapses with two HRP-labeled dendrites(D1, D2)and one negative spine (S) (b). BDA-labeled terminal makes asymmetric synapse with neuronal cell body of HRP-labeled premotor neurons (Soma) (c). One BDA-labeled terminal (T1) and one BDA-negative terminal (T2) synapse simultaneously with dendrite of HRP-labeled premotor neurons, and empty-arrowhead indicates the dense-core vesicle in the negative terminal (d). The arrowheads indicate postsynaptic specializations; N means nucleus of the cell body. a–d are taken from gigantocellular reticular formation, the dorsal medullary reticular formation, parvicellular reticular formation and supratrigeminal nucleus, respectively. Scale bars = 0.3 µm in a; 0.45 in b and d and 0.5 µm in c.

**Figure 7:**
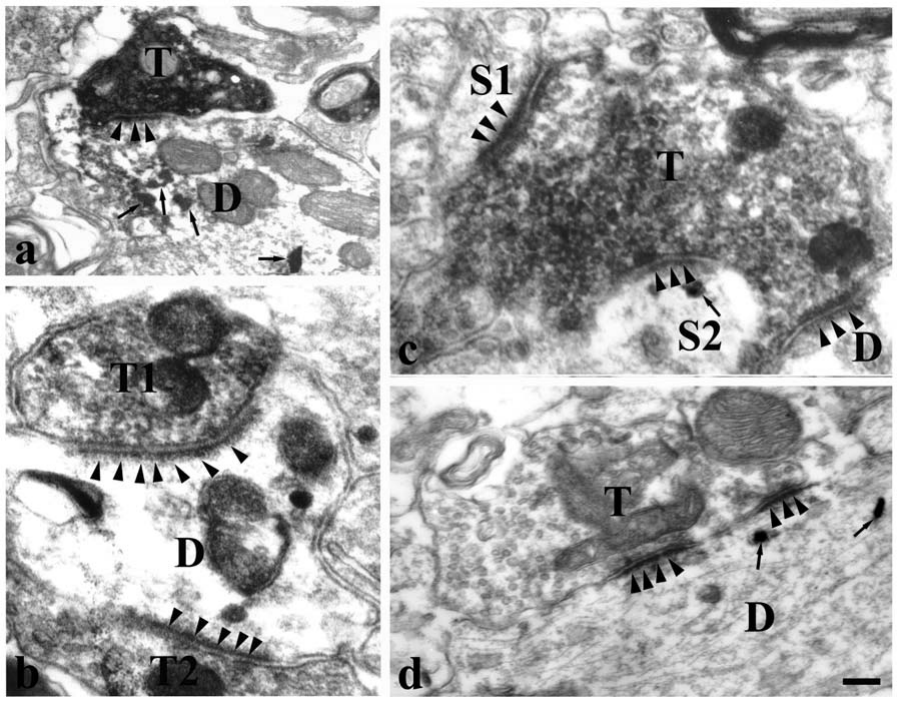
Electron micrograph showing BDA-labeled terminals and WGA-HRP-labeled soma and dendrites in the brainstem of a rat. WGA-HRP was injected into the XII of the rat and BDA into its ipsilateral Vc. BDA-labeled S-type terminals (T) containing spherical vesicles make asymmetric synapses with dendrites (D) of HRP-labeled premotor neurons (a). Two BDA-labeled terminals (T1, T2) make asymmetric synapses simultaneously with dendrite of HRP-labeled premotor neuron (b). A BDA-labeled terminal makes asymmetric synapses simultaneously with one HRP-labeled spine (S2), one negative dendrite (D) and one negative spine (S1) (c). One negative terminal makes asymmetric synapse with HRP-labeled dendrite (d). The arrowheads indicate postsynaptic specializations. a and b are taken from the dorsal medullary reticular formation, c and d from the parvicellular reticular formation. Scale bars = 0.5 µm in a; 0.35 µm in b and c and 0.45 µm in d.

WGA-HRP-labeled premotor neurons were detected by the presence of highly electron-dense clumps of crystalline, sometimes amorphous punctual structure in the cytoplasm of the dendrites and somata (Figs. 6, 7). BDA-labeled axonal terminals were identified ultrastructurally by the presence of predominantly homogeneously distributed, fine granular, electron dense cytoplasmic reaction product (Figs. 6, 7) which was located in the cytoplasm and attached to the membranes of synaptic vesicles and mitochondria. Synaptic vesicles were characterized by electron-lucent flattened, spherical or ovoid structures which were located inside the labeled boutons.

The synaptic contact of BDA-labeled boutons with dendrites or somata of WGA-HRP-labeled neurons were observed, and these synapses were mainly distributed in PCRt, MdD, MdV, Vsup and PBN. The synapses were counted in the PCRt of five sections. Quantificative analysis showed 73% of BDA-labeled boutons (88 among 120) made synapses with dendrites of WGA-HRP-labeled premotor neurons, while 27% (32/120) formed axo-somatic synapses. The vast majority of synapses were asymmetric in which the presynaptic elements contained small, clear and spherical synaptic vesicles, and the postsynaptic membrane was overtly thickened and specialized (Figs. 6, 7).

In addition, there were also some synapses between BDA-labeled boutons and dendrites or somata without HRP product, or between negative boutons and HRP-labeled dendrites or somata of premotor neurons. Some negative axonal terminals included dense-core particles (Fig. 6d).

## Discussion

In our present study, the direct connection of projection fibers from Vc with the premotor neurons which sent their axons to the VII or XII, and both to the VII and XII by axon collaterals in the brainstem was examined using tracer tracing method under both light and electron microscopic levels. In addition, we detected that some promoter neurons in which Fos protein-like immunoreactivity was induced by subcutaneous injection of formalin into the lips apposed to the Vc projecting fibers. These results indicate that those premotor neurons which sent their axons to the VII or/and XII are involved in local orofacial reflex circuits.

### 1. Local orofacial nociceptive reflex circuit in the brainstem

The premotor neurons projecting to VII or XII had the same distribution pattern and were mainly located in the brainstem RF, including PCRt, MdD. The previous morphological experiments had shown that the last-order premotor neurons projecting to the VII or XII were mainly distributed in the PCRt and MdD [15], [17], [24]–[27]. Our findings were in agreement with those in previous publications. In our present experiment, FG-labeled neurons in XII were observed after FG injection into VII. In the previous reports, besides the larger α motoneurons in the XII, there were also small interneurons projecting to other motonuclei [27]–[29]. Some researchers suggested that these interneurons in XII were involved in the modulation of both tongue and facial muscle movements, and thus played an important role in keeping the harmony of the tongue and the facial muscles activities.

To confirm whether these premotor neurons receive afferent projecting from the Vc, we observed the close appositions between the BDA-labeled fibers and FG-labeled premotor neurons by CLSM as well as the synaptic contact between these two components by electron microscope.

The Vc has traditionally been considered as an essential brainstem relay for the orofacial peripheral nociceptive information transmission. The previous studies also demonstrated that the Vc may be a critical element in the neural pathways underlying ONR [12], [13], [30]. Electrophysiological and behavioral observations indicated that transaction of the Vc or ibotenic acid-destroyed Vc resulted in markedly reduced nociception-evoked reflexible EMG responses of the digastric muscle and masseter, and in an increased nociceptive jaw-opening reflex threshold but no similar effects occurred if ibotenic acid was injected into the Vo. Moreover, the Vc neurons had dense projections to the RF, trigeminal sensory complex, PBN and raphe nuclei complex. Electrophysiological study indicated that there were one or more premotor neurons in the pathways of the nociceptive transmission from Vc to brainstem motonuclei [6] and that these premotor neurons were likely to be distributed in PCRt, MdD, MdV, and the trigeminal sensory complex. Our present study showed that the synapses between the dendrites or somata of FG-labeled premotor neurons and BDA-labeled axonal terminals were mainly located on the aforementioned nuclei. Besides, the trigeminal nociceptive afferents synapsed upon premotor neurons projecting to the VII or XII and they might be involved in neuronal networks for the local circuits of the orofacial nociceptive reflex in the brainstem, which was in agreement with the previous electrophysiological studies [6], [31].

A proto-oncogene *c-fos* is one of the most extensively investigated immediate early genes in the sensory nervous systems. It is well known that the peripheral nociceptive stimulation can induce the Fos-protein expression in some special neurons of the central nerve system. In order to further identify that the brainstem premotor neurons were involved in nociceptive information transmission in our present studied pathways, we used Fos expression as the marker of nociceptive neurons by injecting formalin into the peri-oral region of rats subcutaneously and corroborated whether the premotor neurons receiving the Vc projection participated in the nociceptive information transmission to the motoneurons and intervening in the accomplishment of the NR. Our present results show that some premotor neurons projecting to the XII or VII express the Fos-protein after peripheral nociceptive stimulation, which further provides morphological evidence for the existence of the brainstem local circuits for the orofacial nociceptive reflexes.

**Figure 8:**
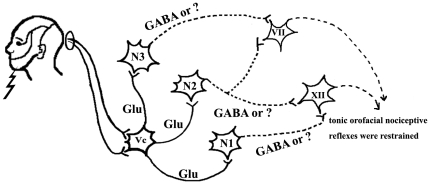
Schematic representation of one possibly neuronal networks model of ONR. The orofacial nociceptive afferents converge onto the neurons of the Vc. The Vc projecting (glutamatergic excitatory axons) act on the motoneurons of the VII or XII via inhibitory premotor neurons (N1, N2 and N3) in the brainstem, and then the tonic orofacial nociceptive reflexes are decreased. Real lines represent excitatory, and the dotted lines are inhibitory. ? represents other inhibitory neurotransmitters.

The synaptological characteristics of projecting from the Vc to premotor neurons were examined by electron microscopy. Our present ultrastructural observations demonstrated that the axonal boutons from the Vc were S-type containing small, clear and spherical synaptic vesicles, and predominantly formed asymmetric synapses with the dendrites of the premotor neurons projecting to the VII or XII. This result suggested that the innervations of the Vc neurons to the premotor neurons was excitatory, which is consistent with our previous study [15]. In the experiment, BDA-labeled axonal boutons made asymmetric synapses on the GABAergic neurons in the Vsup after BDA injection into Vc of the GAD67-GFP knock-in mouse. And some of these BDA-labeled axonal boutons were immunoreactive for the vesicular glutamate transporter 2 (VGluT2), a kind of markers for glutamatergic axonal terminals (data not shown). Meanwhile, most GABAergic neurons in the Vsup were premotor neurons which gave off their axons to motonuclei such as the Vmo. It is presumably clear from the previous and present studies that the glutamatergic axonal terminals of the Vc apposed to the GABAergic premotor neurons of the VII or XII, by which the tonic NR induced by the orofacial nociceptive stimulation might be modulated (Fig. 8). However, further morphological and electrophysiological studies are needed to confirm this postulation.

There are also some BDA-negative axonal terminals making asymmetric synapses on the dendrites of premotor neurons contain dense-core vesicles, which suggests these boutons are likely to contain substance P or enkephalin. In terms of neurochemical properties of projections from the premotor neurons to cranial motoneurons, previous studies indicated that some neuropeptides like substance P and enkephalins existed in the projecting terminals [32], [33]. However, it is unclear whether the brainstem premotor neurons are also innervated by the projecting axonal terminals containing substance P.

### 2. Premotor neurons which are involved in the orofacial nociceptive reflexes project simultaneously to the VII and XII by sending bifurcating axons

Oral motor behaviors including feeding, mastication, swallowing, vocalization and respiration are complex movements. The precise coordination of different orofacial muscles and motor nuclei is necessary for executing the complex function. For example, during chewing, the masser begins rhythm activities concomitantly with the movement of tongue [34]–[36]. Besides, facial muscles, especially the buccal muscle which is an essential ingredient of the oral cavity wall, involve the constitution of the muscle pathway for food or liquid and also take part in chewing movement. However, these muscles are innervated by different motor nuclei in the brainstem. How do the different muscle movements keep in agreement? Why does the nociceptive stimulation induce NRs of orofacial muscles administrated by distinct motor nuclei or subnuclei in the brainstem?

Morphological studies have revealed that the distribution of the premotor neurons of Vmo, VII and XII is overlapping in the brainstem [17], [24]. In addition, some researchers have observed the distribution of double-projecting premotor neurons in rats by using a fluorescent retrograde double-labeling method [18], [19], [27], [28], [37]. In the present study, we also checked the distribution of the premotor neurons which send collateral projections to both the VII and XII after FG and TMR injections. Our findings are discrepant from those Dauvergne et al (37), especially at the level of pons, which may caused by the different retrograde tracers injection and different injection boundaries.

The doubly labeled premotor neurons were mainly distributed in the brainstem, especially in the RF and probably subserved the coordination of orofacial movements required for the complex oral motor behaviors. Furthermore, physiological studies showed that stimulating various trigeminal nerve branches could elicit reflex responsiveness of different groups of brainstem motoneurons [38], [39]. Based on these experiments, we hypothesized that shared brainstem circuits could transmit trigeminal afferent information (such as nociceptive information) to more than one motor nuclei, influence the responses of different motoneurons and play a role in orofacial coordinated muscle movements. However, there is lacks enough morphological evidence to prove the existence of these common brainstem circuits until now. Therefore, in the present experiments, the tracer tracing method was performed to examine the existence of the premotor neurons which received the nociceptive afferents coming from the Vc and then projected to both the VII and XII, simultaneously.

After FG injection into the VII, TMR into XII and BDA into Vc, some FG/TMR double-labeled premotor neurons, receiving projections from Vc and sending their axonal collaterals simultaneously to the VII and XII, were mainly observed in the pontomedullary RF, such as the ventral part of Vmo, PCRt, MdD and MdV. Dauvergne [37] has reported that the RF neurons of the trigemino-reticulo-facial pathways involved in blinking mechanisms also project to the XII, which was coincident with our present result. However, Dauvergne's research only observed roughly the contact between the trigeminal primary afferents and the double-labeled neurons in the RF but did not get involved in the functional significance of such dual facial-hypoglossal projections. An important finding of our present study is that some double-labeled neurons which project to both the VII and XII expressed Fos protein after formalin was injected into the lips. It is tempting to prove that these double labeled premotor neurons were involved in the synchronous activities of the facial and hypoglossal motoneurons after nociceptive stimulation.

According to our experiments, not so many double-labeled premotor neurons closely appose with Vc fibers, which may be related with the limitation of the tracer-tracing method we used. The retrograde tracer FG and WGA-HRP cannot be trans-synaptically transported, so the bi-synapse pathways can only be traced [25], [26], [40]–[43], though most brainstem nociceptive reflexes are mediated by multi-synaptic pathways in the brainstem [6].

In conclusion, our study has corroborated the distribution of the premotor neurons which are involved in the brainstem local circuits of the nociceptive reflexes and has demonstrated that the orofacial nociceptive afferents axon terminals synapse upon the premotor neurons of the VII and XII under the electron microscope. Moreover, we provide some morphological evidence for existence of the premotor neurons projecting simultaneously to both the VII and XII by the axonal collaterals. These premotor neurons may play an important role in harmonizing different orofacial muscles movements and accomplishing orofacial complex nociceptive reflexes.
